# Carbon-Based Nanomaterials from Biopolymer Lignin via Catalytic Thermal Treatment at 700 to 1000 °C

**DOI:** 10.3390/polym10020183

**Published:** 2018-02-13

**Authors:** Xuefeng Zhang, Qiangu Yan, Jinghao Li, I-Wei Chu, Hossein Toghiani, Zhiyong Cai, Jilei Zhang

**Affiliations:** 1 Department of Sustainable Bioproducts, Mississippi State University, Mississippi State, MS 39762, USA; njfuxf@gmail.com; 2 U.S. Department of Agriculture, Forest Service, Forest Products Laboratory, Madison, WI 53726, USA; yanqiangu@gamil.com (Q.Y.); csuftljh@gmail.com (J.L.); 3 Institute of Imaging and Analytical Technology, Mississippi State University, Mississippi State, MS 39762, USA; ic120@msstate.edu; 4 Dave C. Swalm School of Chemical Engineering, Mississippi State University, Mississippi State, MS 39762, USA; hossein@che.msstate.edu

**Keywords:** biopolymer, kraft lignin, graphitic-carbon-encapsulated iron nanoparticles, temperature, amorphous carbon, carbonaceous gases

## Abstract

We report the preparation of carbon-based nanomaterials from biopolymer kraft lignin via an iron catalytic thermal treatment process. Both the carbonaceous gases and amorphous carbon (AC) from lignin thermal decomposition were found to have participated in the formation of graphitic-carbon-encapsulated iron nanoparticles (GCEINs). GCEINs originating from carbonaceous gases have thick-walled graphitic-carbon layers (10 to 50) and form at a temperature of 700 °C. By contrast, GCEINs from AC usually have thin-walled graphitic-carbon layers (1 to 3) and form at a temperature of at least 800 °C. Iron catalyst nanoparticles started their phase transition from α-Fe to γ-Fe at 700 °C, and then from γ-Fe to Fe_3_C at 1000 °C. Furthermore, we derived a formula to calculate the maximum number of graphitic-carbon layers formed on iron nanoparticles via the AC dissolution-precipitation mechanism.

## 1. Introduction

Lignin is the most abundant aromatic biopolymer on Earth and is available in large quantities from wood-pulping and bio-ethanol industries [1]. Extensive efforts have been made to convert lignin to fuels and chemicals [2,3], but the high processing cost is the major challenge for widespread application of these technologies. Lignin contains more than 60% carbon, which is a higher content than that found in cellulose and hemicellulose (~40%), and can be a good alternative green carbon source for the production of carbon-based nanomaterials (CBNs) [4,5,6].

CBNs with a graphitic framework, including carbon nanotubes, carbon-encapsulated metal nanoparticles, and graphene, have attracted great interest in past decades because of their applicability in numerous areas. For instance, carbon-encapsulated metal nanoparticles have been reportedly used as catalysts for Fischer-Tropsch synthesis [7,8] and as drug deliver carrier for biomedical applications [9] owing to their unique catalytic and magnetic properties [10,11,12]. Chemical vapor deposition (CVD) is the most widely used method for CBNs synthesis, in which a carbonaceous gas or vapor passes through a tubular reactor zone and dissociates to carbon atoms that add onto the catalyst/substrate to form a graphitic-carbon network. Although the CVD process produces CBNs with high purity, high selectivity, and less defects, the high synthesis cost and the use of non-renewable carbon precursors limits the process scalability [7]. 

Recently, a catalytic thermal treatment synthesis process has been developed for converting renewable biomass and biopolymers to CBNs, where renewable biomass and biopolymers such as sawdust, saccharides, lignosulfonate, and acetic acid lignin are thermally treated at 800 to 1200 °C using transition metal particles and various metal salts as catalysts [13,14,15,16]. Although the catalytic thermal treatment of biopolymers is a green and promising route for the production of CBNs, the low purity of the products and the difficulty of regulating the formation process remain problems, because these biopolymers show heterogeneity in both structure and composition [13].

The dissolution-precipitation theory commonly explains the formation mechanism of CBNs in a catalytic thermal treatment process. During this process, polymers first decompose to amorphous carbon (AC), after which AC, as the solid carbon source, dissolves in and precipitates out of the catalyst [16,17,18,19,20,21]. The dissolution and precipitation of AC is affected by the thermal treatment temperatures. Meanwhile, heating biopolymers can generate various carbonaceous gases [4,22,23] which could also participate in the formation of CBNs with the presentation of transition metals as catalysts. Therefore, a deep understanding of the biopolymers catalytic thermal treatment process is critical in order to regulate the formation of CBNs.

There is no report found in the investigation of how carbonaceous gases and AC as a carbon source participate simultaneously in the formation of CBNs during the catalytic thermal decomposition of a solid carbon source, particularly lignin. In this paper, kraft lignin was promoted with iron nitrate and thermally treated from 700 to 1000 °C. The phase change of the iron catalyst with temperature was studied by X-ray diffraction (XRD), and the morphology and structure of as-synthesized CBNs were characterized using transmission electron microscopy (TEM).

## 2. Materials and Methods

### 2.1. Materials

Kraft lignin (KL) supplied by Domtar Corp. (Plymouth, NC, USA) was used as a carbon source. The KL ash content measured in our lab was 0.53% [1]. Iron nitrate nonahydrate (Fe(NO_3_)_3_·9H_2_O, 98% purity), from Sigma-Aldrich (St. Louis, MO, USA), was the source of the iron catalyst.

### 2.2. Preparation of CBNs

Lignin-Fe precursor (KL/Fe) was prepared through impregnating 40 g of oven-dried lignin into 0.27 mol/L iron nitrate solution followed by evaporating water in a 120 °C oil-bath and oven-drying the precursor at 105 °C [4]. The oven-dried KL/Fe precursor contains lignin, iron, and nitrogen compounds. The weight ratio of iron and nitrogen compounds to oven-dried lignin was 7.50% and 5.08%, respectively (the stoichiometry of KL/Fe preparation is shown in Table S1 in the Supplementary Materials). Four grams of KL/Fe precursor was then loaded into two porcelain boats (each holds ~2 g), after which the two boats were inserted into an in-quartz tube furnace reactor and heated (ramping rate was 20 °C/min) under atmospheric pressure with an argon gas atmosphere (99.99%, 1.8 L/min). Evaluated temperature levels were 700, 750, 800, 900, and 1000 °C, and products were labeled as KL/Fe-X, where X represents the temperature level. After maintaining the rated temperature for 1 h, the furnace was turned off and allowed to cool to ambient temperature under an argon atmosphere. The thermal treatment yields (*Y*, %) and carbon yields (*Y*_c_, %) were calculated using the following equations:

*Y*, % =(*W*_1_/*W*_0_) × 100% (1)

*Y*_c_, % = [(*W*_1_ − *W*_Fe_ − *W*_Ash_)/(*W*_0_ − *W*_Fe_ − *W*_N_ − *W*_Ash_)] × 100% (2)

where *W*_1_ is the solid materials weight after thermal treatment (g), *W*_0_ is the KL/Fe precursor weight, *W*_Fe_ is the weight of iron in the KL/Fe precursor, *W*_Ash_ is the weight of ash in the lignin, and *W*_N_ is the weight of nitrogen compounds in the KL/Fe precursor. 

### 2.3. Characterization

The distribution of gas compounds (i.e., CH_4_, CO, CO_2_, and H_2_) from the thermal treatment of KL/Fe was analyzed on an Autochem II 2920 chemisorption system (Micromeritics Instrument Corp., Norcross, GA, USA). Approximately 5 g of KL/Fe sample was thermally treated in the chemisorption system at the temperature range from 50 to 1000 °C at a ramping rate of 10 °C/min under a nitrogen atmosphere. The chemical composition and morphology of KL/Fe-X samples were characterized by an Ultima3 XRD diffractometer (CuKα radiation, Rigaku, The Woodlands, TX, USA) and a 2100-high-resolution TEM (JEOL, Peabody, MA, USA), respectively. 

## 3. Results

### 3.1. Yield

The thermal treatment yield (*Y*, %) and carbon yield (*Y*_c_, %) of KL-X samples slightly decreased from 49.28% to 48.55% and from 47.98% to 46.63% as the temperature increased from 700 to 1000 °C (Table 1). In addition to using the described furnace system, the treatment of KL/Fe was also conducted by thermogravimetric (TG) analysis; the mass loss and mass loss rate curves are shown in Figure S1 (in Supplementary Materials).

### 3.2. XRD

**Figure 1:**
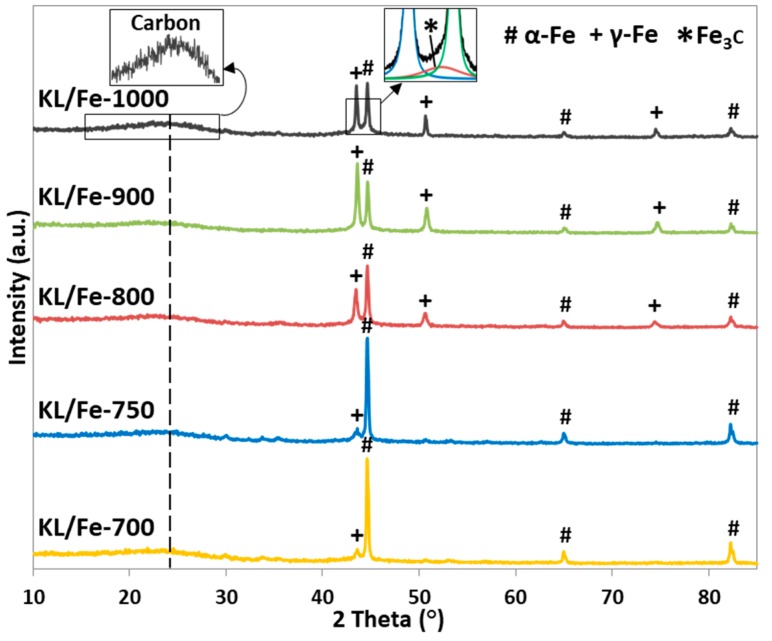
X-ray diffraction (XRD) patterns of KL/Fe-X samples.

The XRD patterns of KL/Fe-X samples (Figure 1) show all detected iron- and carbon-related diffraction peaks. Peaks appearing at 44.6°, 65.0°, and 82.3° correspond to α-Fe [24], while peaks at 43.6°, 51.0°, and 75.1° are related to γ-Fe (pdf#98-000-0258). Peaks at 24° and 44.4° correspond to the carbon (002) and Fe_3_C (013) reflections, respectively [4,24]. In general, as the temperature increased from 700 to 900 °C, the diffraction intensity of α-Fe decreased while the γ-Fe increased, and as the temperature further increased to 1000 °C, the γ–Fe diffraction intensity (2θ = 43.6°) decreased, while the Fe_3_C intensity appeared. The existence of γ–Fe starting at 700 °C indicated that the γ–Fe was somehow shielded or blocked in the carbon matrix, preventing the phase transformation of γ-Fe to α-Fe and Fe_3_C [25,26] at ambient conditions, because at ambient conditions γ-Fe is thermodynamically unstable and normally transforms to α-Fe and Fe_3_C spontaneously [17]. The iron phase shifting from α-Fe to γ-Fe started at 700 °C, which is lower than the bulk iron phase transformation (α-Fe to γ-Fe) temperature of 727 °C [27]. This can be explained by the size effect from nanosized iron [28]. The continuous phase shifting between α-Fe to γ-Fe as the temperature increased from 700 to 900 °C, and Fe_3_C appearing at 1000 °C indicated that the iron phase transformation occurred through constant carbon dissolution [27].

### 3.3. TEM

**Figure 2:**
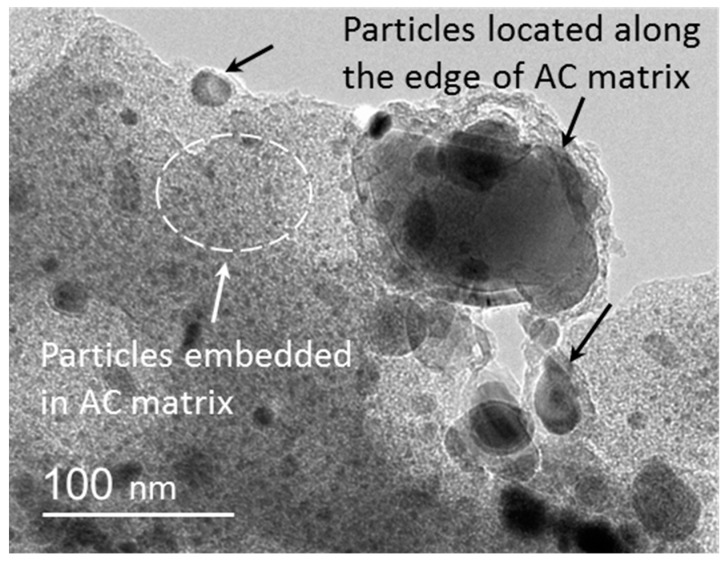
A typical image of KL/Fe-X samples shows that nanoparticles embedded in the amorphous carbon (AC) matrix had diameters of less than 20 nm, while nanoparticles located along the edge of the AC matrix had sizes ranging from 20 to 200 nm.

Figure 2 is a typical TEM image of KL/Fe-X samples, showing the distribution of different sizes of nanoparticles in the AC matrix. In general, the majority of these particles was embedded in the AC matrix and had diameters of less than 20 nm, and only a small amount of particles located along the edge of the AC matrix had diameters ranging from 20 to 200 nm.

**Figure 3:**
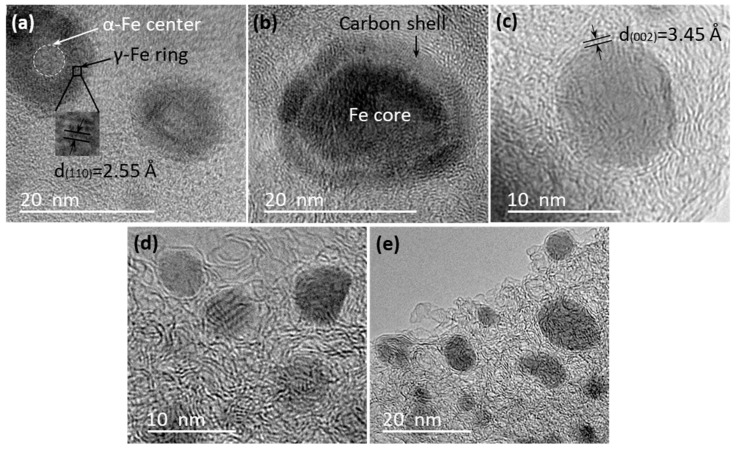
High-magnification TEM images of the iron nanoparticles embedded in the AC matrix in KL/Fe-700 (**a**), -750 (**b**), -800 (**c**), -900 (**d**), and -1000 (**e**) samples.

In order to study the structure and morphology differences between the small and large nanoparticles, a close look at the TEM images of individual particles was taken. High-magnification TEM images indicated that there were obvious differences in the micro-morphology of the small nanoparticles that were embedded in AC matrix at different temperatures. Figure 3a shows that the small nanoparticles in sample KL/Fe-700 had an α-Fe core (light color) and a γ-Fe ring (dark color) structure. The γ-Fe ring was confirmed by a measured interlayer space of 2.55 Å (corresponding to the (110) lattice planes of γ-Fe). This observation corresponds to the XRD results and confirms that the formation of γ-Fe through the AC diffusion into α-Fe started at 700 °C, because γ-Fe was not detected at 600 °C in our previous study [4]. Iron nanoparticles embedded in the AC matrix of KL/Fe-750 sample were encapsulated by carbon shells (Figure 3b). Huo et al. [24] reported a similar carbon-shelled iron core structure by the thermal treatment of aromatic heavy oil and ferrocene at 480 °C under pressure. Lignin contains about 30% oxygen, which requires a higher formation temperature for carbon-shelled iron core structure than aromatic heavy oil. No clear graphitic-carbon structure was observed in these carbon shells in samples heated up to 750 °C. Figure 3c shows a thin-walled graphitic-carbon-encapsulated iron nanoparticle (GCEIN) observed in the AC matrix of the KL/Fe-800 sample, evidenced by a measured (002) interlayer space of 3.45 Å (corresponding to the turbostratic stacking structure [4]). Figure 3d,e show similar thin-walled GCEINs observed to be embedded in the AC matrix of KL/Fe-900 and -1000 samples, respectively. These thin-walled GCEINs are usually composed of one to three graphitic-carbon layers and the size of the thin-walled GCEINs were less than 20 nm. These TEM observations indicate that there is a minimum temperature of 800 °C that is required for the formation of thin-walled GCEINs.

**Figure 4:**
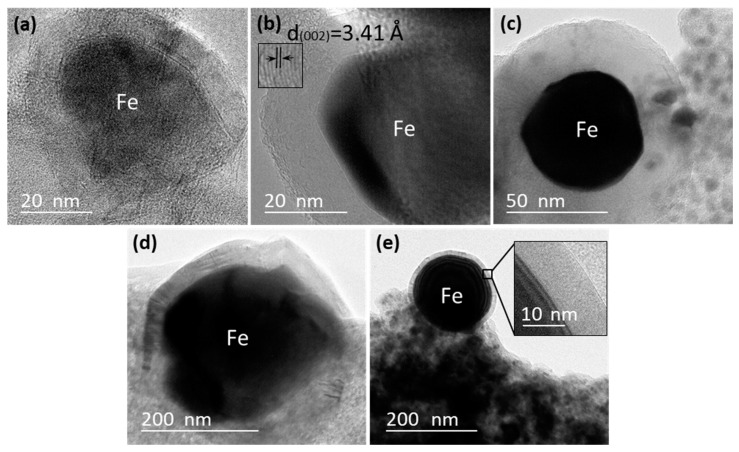
High-magnification TEM images of KL/Fe-700 (**a**), -750 (**b**), -800 (**c**), -900 (**d**), and -1000 (**e**) samples showed that the iron nanoparticles located along the edge of the AC matrix had 10 to 50 layers making up thick-walled graphitic-carbon shells.

High-magnification TEM images (Figure 4) showed that the iron nanoparticles located along the edge of the AC matrix were encapsulated by thick-walled graphitic-carbon layers ranging from 10 to 50. The diameter of these thick-walled GCEINs ranged from 20 to 200 nm. Similar thick-walled GCEINs were reported in the literature related to the thermal treatment of woody biomass at the temperature range from 700 to 1000 °C using iron catalysts [16,20,21,22].

## 4. Carbon Source for GCEINs Formation: AC or Carbonaceous Gases

**Figure 5:**
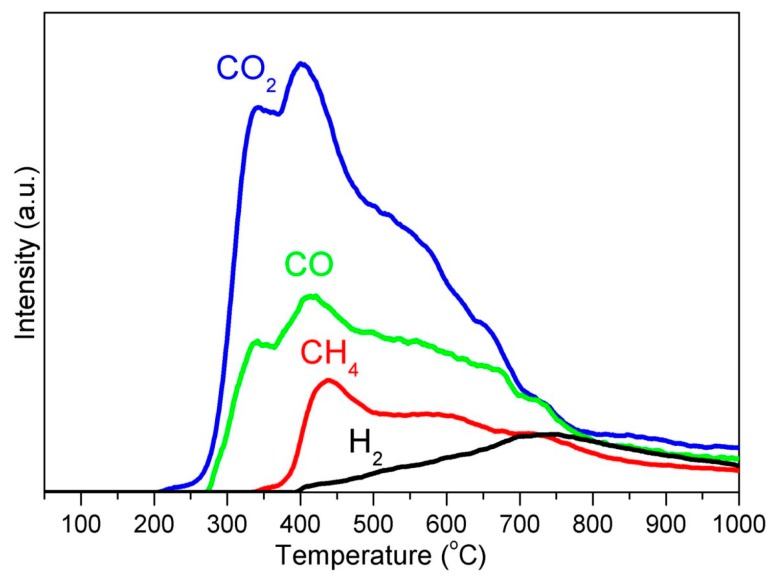
Gas evolution profiles of during thermal treatment of the KL/Fe precursor.

During the thermal treatment, iron nitrate was first decomposed to iron oxide nanoparticles at 300 °C, then become converted into α-Fe at 600 °C [4], followed by the phase transition from α-Fe to γ-Fe at 700 °C, and then γ-Fe to Fe_3_C at 1000 °C. Meanwhile, the thermal decomposition of the KL/Fe precursor generated solid AC and various carbonaceous gases such as CH_4_ and CO (Figure 5). Both AC and carbonaceous gases can react with an iron catalyst at high temperatures to form CBNs. TEM observations revealed two pieces of information: (1) when iron nanoparticles are embedded in the AC matrix (meaning that the iron has no contact with carbonaceous gases), the GCEINs have thin-walled graphitic-carbon shells (1–3 layers); and (2) when iron nanoparticles are located along the edge of the AC matrix (meaning that the iron has contact with carbonaceous gases), the GCEINs have thick-walled graphitic-carbon shells (10–50 layers). Therefore, the formation of thin-walled GCEINs was achieved through a solid carbon dissolution and precipitation process, i.e., AC acted as the carbon source. Conversely, the formation of thick-walled GCEINs was through a CVD process, i.e., carbonaceous gases acted as the carbon source. In order to verify our inference that carbonaceous gases affect the formation of GCEINs, a simple experiment was designed to compare the yield of KL/Fe-1000 located in an argon gas inflow side with that in an outflow side (detailed information is shown in the Supplementary Materials). The statistical analysis indicated that the yield of KL/Fe-1000 in the outflow side was significantly higher than that in the inflow side, which further confirmed that carbonaceous gases affect the formation of GCEINs.

There is a question about thin-walled GCEINs: why can only a few layers of a graphitic-carbon shell be formed when AC acts as the carbon source? We think that this phenomenon is due to the isolation effect; once the dissolved carbon precipitated from the iron nanoparticles and formed the graphitic-carbon layer, the dissolution and precipitation processes were self-terminated because the graphitic-carbon layer had prevented the dissolution of AC into iron. In other words, the formation of thin-walled GCEINs from AC dissolution and precipitation was a one-off process. Therefore, the number of graphitic-carbon layers depended on the maximum concentration of carbon in iron.

**Figure 6:**
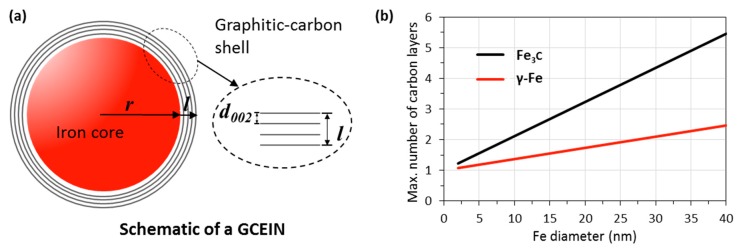
(**a**) Schematic of a graphitic-carbon-encapsulated iron nanoparticle (GCEIN) model and (**b**) estimated maximum number of graphene-layers vs the diameter of iron nanoparticles.

Figure 6a illustrates the proposed GCEIN model with the assumption that an iron nanoparticle is a perfect sphere. The number of graphitic-carbon layers, *N*, formed around an iron nanoparticle can be estimated using Equation (3):

(3) $$N=\frac{l}{d_{002}}+1$$

where *l* is graphitic-carbon thickness (Å) and *d*_002_ is the interlayer space of the graphitic-carbon (3.45 Å, Figure 3c).

The graphitic-carbon thickness can be estimated using Equation (4):

(4) $$l={\left(\frac{3}{4}\times \frac{V_{g}}{\pi }+r^{3}\right)}^{\frac{1}{3}}-r$$

where *V*_g_ is graphitic-carbon volume (nm^3^) and *r* is iron nanoparticle radius (nm).

The graphitic-carbon volume can be estimated using Equation (5) with the numerator representing the weight of carbon dissolved in an iron nanoparticle:

(5) $$V_{g}=\frac{\frac{4}{3}\times \pi \times r^{3}\times \rho_{Fe}\times C_{wt}}{\rho_{g}}$$

where *ρ*_g_ is graphitic-carbon density (2.09 g/cm^3^), *ρ*_Fe_ is iron density (7.84 g/cm^3^), and *C_wt_* is the weight concentration of carbon in iron (wt %).

Figure 6b illustrates the maximum number of graphitic-carbon layers around an iron nanoparticle estimated using Equation (3) with the assumption of the maximum weight concentrations of carbon in iron (2.14 and 6.70 wt % for γ-Fe and Fe_3_C [27], respectively). The estimated maximum numbers of graphitic-carbon layers were 1.2 and 1.7 for γ-Fe core diameters of 5 and 20 nm, respectively, while the layer numbers were 1.6 and 3.3 for the Fe_3_C core diameters of 5 and 20 nm, respectively. The calculation results are in agreement with the TEM observations and explained why only few-layer graphitic-carbon shells can be formed when AC acts as the carbon source.

**Figure 7:**
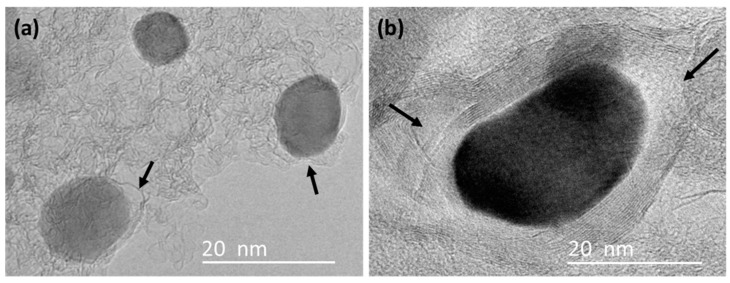
TEM images of (**a**) thin-walled GCEINs and (**b**) thick-walled GCEINs in sample KL/Fe-800. The structural defects in the graphitic-carbon shells are indicated by arrows.

There is another question about thick-walled GCEINs: why there is no isolation effect when carbonaceous gases act as the carbon source? We think this phenomenon is due to the structural defects of graphitic-carbon shells, as shown in Figure 7 (indicated by arrows). These defects can act as channels for carbonaceous gases to cross the graphitic-carbon shells and react with the iron catalyst to form more graphitic-carbon layers.

## 5. Conclusions

In this study, GCEINs were prepared through the iron catalytic thermal treatment of kraft lignin. The formation of thin-walled GCEINs embedded in AC was based on the dissolution and precipitation mechanism of amorphous carbon acting as the solid carbon source. The formation of thick-walled GCEINs appearing along the edge of the AC matrix was based on the CVD process, in which carbonaceous gases served as the carbon source. Moreover, we derived a formula for the calculation of the maximum number of graphitic layers formed on iron nanoparticles via the AC dissolution-precipitation mechanism. This formula can be applied to various GCEIN synthesis conditions using polymers as carbon precursors for the determination the role of carbonaceous gases and AC in GCEIN formation.

## Figures and Tables

**Table 1:** Thermal treatment yields (*Y*, %) and carbon yields (*Y*_c_, %) of kraft lignin (KL)/Fe-X samples.

	KL/Fe-700	KL/Fe-750	KL/Fe-800	KL/Fe-900	KL/Fe-1000
*Y*, *%*	49.28	48.96	48.77	48.62	48.55
*Y*_c_, *%*	47.70	47.34	47.13	46.96	46.88

## Supplementary Materials

The supplementary materials are available online at http://www.mdpi.com/2073-4360/10/2/183/s1.
