# Supplementary material for: Carbon-Based Nanomaterials from Biopolymer Lignin via Catalytic Thermal Treatment at 700 to 1000 °C
Source: Polymers (Basel). 2018 Feb 13;10(2):183. doi: 10.3390/polym10020183 (PMC6415029; doi:10.3390/polym10020183)
Supplement: Supplementary file 1 [file polymers-10-00183-s001.pdf]

Carbon-based Nanomaterials from Biopolymer Lignin *via* Catalytic Thermal Treatment at 700 to 1000 °C

Xuefeng Zhang [1], Qiangu Yan [2], Jinghao Li [2], I-Wei Chu [3], Hossein Toghiani [4], Zhiyong Cai [2,*], and Jilei Zhang [1,*]

[1] Department of Sustainable Bioproducts, Mississippi State University, Mississippi State, MS 39762, USA;
[2] U.S. Department of Agriculture, Forest Service, Forest Products Laboratory, Madison, WI 53726, USA;
[3] Institute of Imaging and Analytical Technology, Mississippi State University, Mississippi State, MS 39762, USA;
[4] Dave C. Swalm School of Chemical Engineering, Mississippi State University, Mississippi State, MS 39762, USA;

Supplementary Materials

Table S1. Stoichiometry for KL/Fe precursor preparation

Oven-dried KL (g)	$Fe(NO_3)_3 \cdot 9H_2O$ (g)	Fe (g)	Deionized Water (ml)	Oven-dried KL/Fe precursor (g)	Weight ratio (%)	
					Fe to KL	Nitrogen compound to KL
40.000	21.645	3.000	200	45.030	7.50	5.0

Thermogravimetric analyses

Thermogravimetric analysis was conducted to analysis KL/Fe thermal decomposition behavior. TG experiment was performed on a TGA Q5000 thermo-gravimetric analyzer (TA Instruments, New Castle, DE, USA), 5 mg of KL was first pretreated in the argon (99.99%) flow at 25 ◦C for 5 min, then heated up to 1000 °C at a heating rate of 20 °C/min under nitrogen flow (100 mL min^{-1}). The mass loss (TG) and mass loss rate (DTG) as a function of time during KL/Fe thermal decomposition is shown in Figure S1. Two major peaks are shown in KL/Fe DTG curve, the first one located at 393.4 °C corresponding to decomposition of lignin side chains and functional groups, while the other one located at 746.6 °C probably related to the rearrangement and/or creaking of aromatic rings that was induced by iron phase transformation (α-Fe to γ-Fe, shown in Figure 1). It is worth to note that the mass loss of KL/Fe from TG curve between 750 and 1000 °C was 4.85%, which was much higher than the mass loss (the difference in yield between KL/Fe1000 and KL/Fe750) calculated from the furnace system (0.41%, calculated from Table 1). The inconsistency was because TG curve gave a real-time mass of KL/Fe, while the yield from furnace system was the mass of KL/Fe after 1h treatment [1].

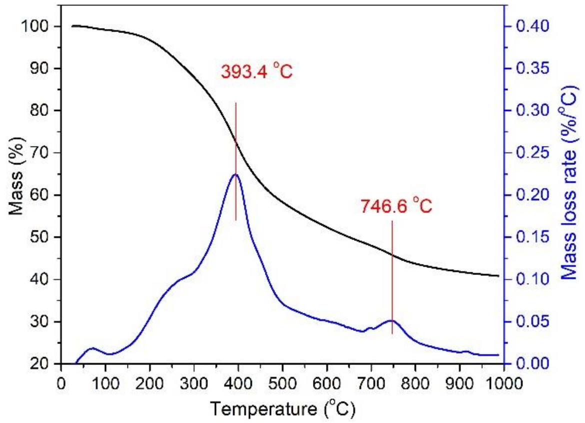

Figure S1. Thermogravimetric analysis of KL/Fe showing TG and DTG curves.

Yield of KL/Fe1000 located in the argon gas inflow side and outflow side

Approximately 4 g of KL/Fe precursor were then loaded into two porcelain boats (each holds ~2 g) placed in the heating zone of a split-hinge 50 mm-quartz tube electric furnace (Lindberg/Blue M 1200. Thermo Scientific™, Pittsburgh, PA, USA) equipped with a temperature controller (Lindberg/Blue UTC 150, Thermo Scientific™, Pittsburgh, PA, USA). The boat No.1 located in argon gas inflow side and the boat No.2 located in argon gas outflow side. After purging argon gas (99.99%) for 15 min to exclude air from the system, the furnace was raised to 1000 °C at a ramping rate of 20 °C/min under atmospheric pressure with an argon gas (99.99%) at a flow rate of 1.8 L/min. The schematic of the furnace system is shown in Figure.S2. Four independent runs were conducted and the yields (Y and Y_c, Table S1) of samples in boat No.1 and boat No.2 were compared using ANOVA. The ANOVA results indicated that the yield of KL/Fe1000 in boat No.1 was significantly higher than that in boat No.2. This was because the carbonaceous gases released from boat No.1 were absorbed by the sample in boat No.2 and formed graphitic nanostructures, as a results, sample in boat No.2 have higher yields.

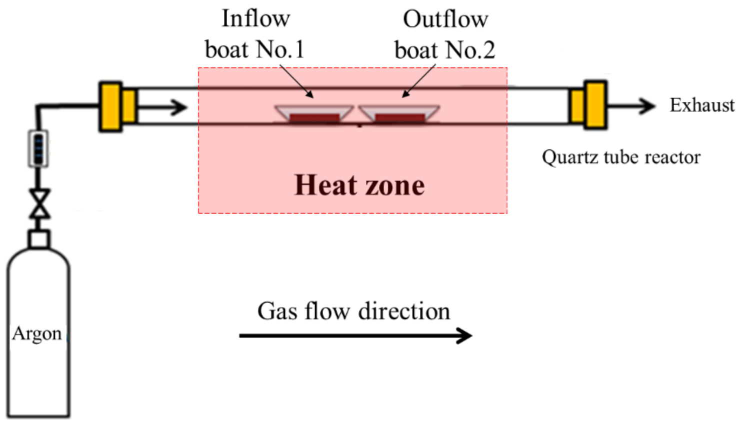

Figure S2. Schematic of the furnace system.

Table S1. Summary of thermal treatment yields and carbon yields of four independent runs

	Thermal treatment yields (Y, %)		Carbon yields (Y_c, %)	
	In flow	Outflow	In flow	Outflow
Run 1	48.25	48.73	46.54	47.08
Run 2	48.58	48.91	46.91	47.28
Run 3	48.23	48.50	46.51	46.83
Run 4	48.45	48.78	46.76	47.14
Average	48.38	48.73	46.68	47.08
COV (%)	0.35	0.35	0.41	0.40
ANOVA	Significant difference P-value = 0.0053		Significant difference P-value = 0.0046	

1. Zhang, X.; Yan, Q.; Leng, W.; Li, J.; Zhang, J.; Cai, Z.; Hassan, E. B. Carbon nanostructure of kraft lignin thermally treated at 500 to 1000 °C. *Materials* **2017**, *10*, 975, doi:10.3390/ma10080975.
